# Supplementary material for: How to Avoid Lower Priority for Smoking Cessation Support Content on Facebook: An Analysis of Engagement Bait
Source: Int J Environ Res Public Health. 2023 Jan 5;20(2):958. doi: 10.3390/ijerph20020958 (PMC9859185; doi:10.3390/ijerph20020958)

Supplementary Materials File S1:

Examples of Facebook post categories.

1. Engagement Bait

“Engagement bait” is a strategy to create Facebook posts that lead people to interact, through likes, shares, comments, and other actions, in order to artificially boost engagement and get greater reach in News Feed.

a. Comment baiting

Asking people to comment with specific answers (words, numbers, phrases, or emojis).

e.g.,

Write your opinion in comment!

The "Tobacco 21" movement aims to raise the age limit for buying tobacco products to 21 years.
This may reduce the rate of smoking among young people.

Should the age limit of 21 be introduced in Hungary?
"Yes!" or "No!"

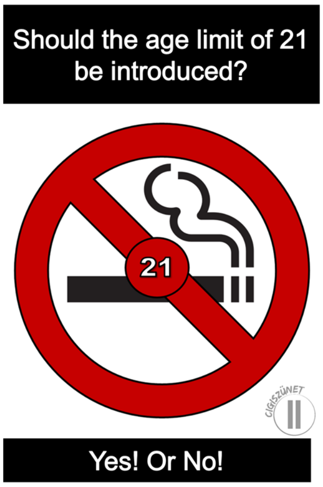

b. Vote baiting

Asking people to vote using reactions, comments, sharing, or other means of representing a vote.

e.g.,

Vote using the reaction buttons!

How do you deal with stress?

With cigarettes, fitness or maybe music?

With "Like", "Love" or maybe "Wow"?

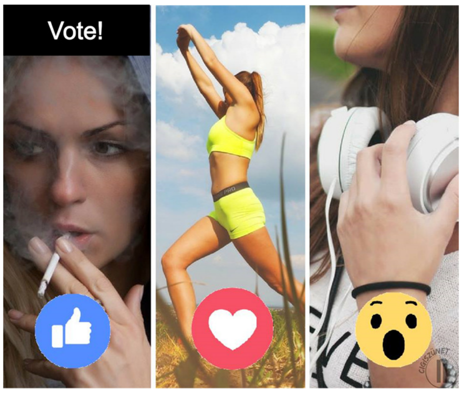

2. Alternatives to Engagement Bait

These asking strategies encourage people to interact without engagement bait. These strategies use questions, rather than instructions.

a. Questions instead of comment baiting

Using open questions about the topic of the given content.

e.g.,

Everyone is motivated by
other things to achieve their goals.

What motivates you
to quit smoking?

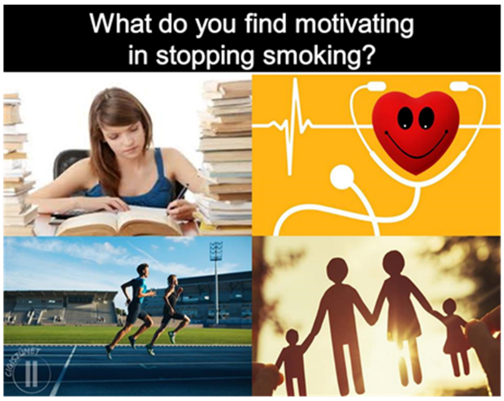

b. Questions instead of react baiting

Highlighting the emotional background of the given content. Using questions about emotions related to the given content.

e.g.,

This face illustrates the initial emotions of smoking cessation.

What about your feelings?

How do you feel in this situation?

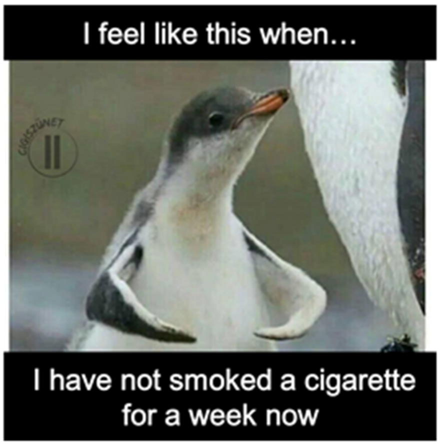

Supplement: Supplementary file 1 [file ijerph-20-00958-s001.zip › Supplementary Materials File S1.pdf]
