# Supplementary material for: How to Avoid Lower Priority for Smoking Cessation Support Content on Facebook: An Analysis of Engagement Bait
Source: Int J Environ Res Public Health. 2023 Jan 5;20(2):958. doi: 10.3390/ijerph20020958 (PMC9859185; doi:10.3390/ijerph20020958)

Supplementary Materials File S2

Examples of the excluded contents.

1. **Non-cessation.**
   Facebook posts which did not support quitting.

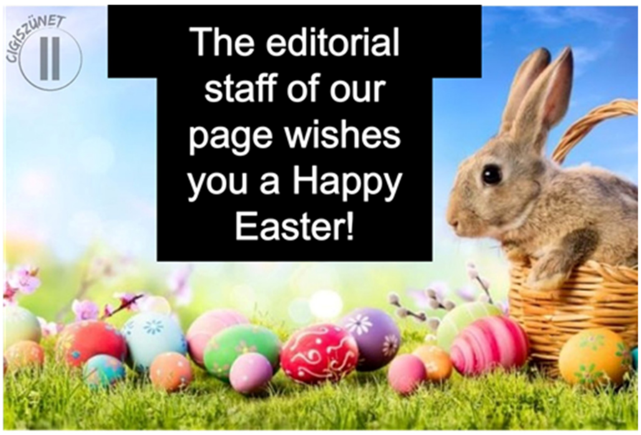

2. **Non-image based.**
   Facebook posts which contained a video or a link only.

   "This is an interesting article about smoking cessation:
   www.facebook.com/cigiszunet"

3. **Paid (boosted).**
   Facebook posts which were promoted by paid advertising after publication.

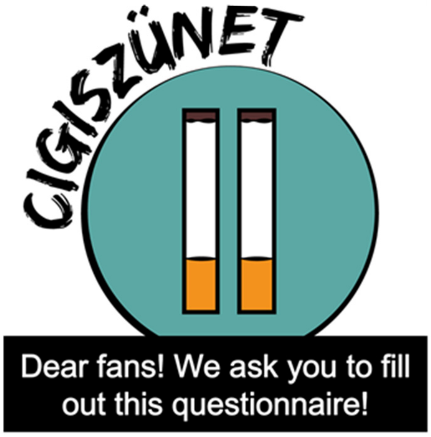

Supplement: Supplementary file 1 [file ijerph-20-00958-s001.zip › Supplementary Materials File S2.pdf]
